# Poststaphylococcal coagulase negative reactive arthritis: a case report

**DOI:** 10.1186/1757-1626-2-9352

**Published:** 2009-12-18

**Authors:** Xhevdet Krasniqi, Sylejman Rexhepi, Masar Gashi, Blerim Berisha, Flora Abazi, Dardan Koçinaj

**Affiliations:** 1 University Clinical Center of Kosova, Rrethi i Spitalit p.n, 10000 Prishtina, Republic of Kosova

## Abstract

We report a case of a 49-year-old patient who developed poststaphylococcal coagulase negative reactive arthritis. The woman presented with constitutional symptoms, arthritis, urinary infection and conjunctivitis. The blood culture was positive for the staphylococcal coagulase negative infection. Erythrocyte sedimentation rate and C-reactive protein were elevated, whereas the rheumatoid factor was negative. Radiographic findings confirmed diagnosis of pleuropneumonia, and one year later of chronic asymmetric sacroileitis. Physicians should be aware of possible reactive arthritis after staphylococcal coagulase negative bacteremia.

## Introduction

Reactive arthritis refers to the acute nonpurulent infection as a complication elsewhere in the body. Human leukocyte antigen B-27 is positive in 60-85% of patients with reactive arthritis but this may also occur in patients who are HLA-B27 negative [1-3]. Post-staphylococcal coagulase negative reactive arthritis is an uncommon disease entity. Coagulase negative staphylococci, although considerably less virulent than *Staphylococcus aureus*, are among the causes of different infections. Some of these species are components of the normal human flora found on the skin, oropharynx and vagina. Coagulase negative species like Staphylococcus epidermidis are increasingly associated with hospital-acquired infections, whereas Staphylococcus saprophyticus causes urinary infections [4,5].

We report a case of reactive arthritis after coagulase negative staphylococcus, which presented with typical clinical and radiographic findings [6].

## Case presentation

A 49-years-old Kosovo Albanian woman presented to our clinic with constitutional symptoms, including fatigue, malaise, fever and weight loss. The musculoskeletal symptoms had acute onset. Arthritis was asymmetric and painful with involvement of the upper and lower extremities such as wrists, the first proximal interphalangeal joint (PIP) of the right hand, knees, ankles, and also the lumbosacral spine.

At the admission, her body temperature was 37.5°C, pulse rate was regular of 80 beats/min and blood pressure was 130/80 mmHg. The patient could not walk without support. There were no oral or genital ulcers and no skin lesions, and neurological findings were characterized with stiffness of the neck. Also, she had conjunctivitis, and cystourethritis resulting from urinary catheter.

**Figure 1:**
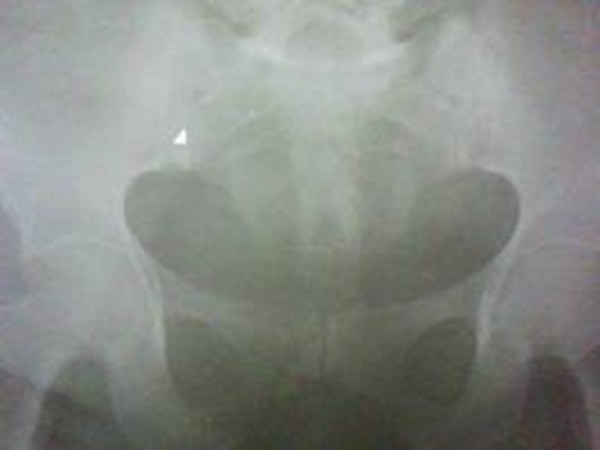
**Anteroposterior radiographs of the pelvis showing asymmetric erosive sacroileitis on the right side**.

Among the laboratory findings, hematology tests showed white blood cell count of 12.2 × 10^3^/mm^3^, red blood cell count of 4.0 × 10^6^/mm^3^, hemoglobin 12.0 g/dl, platelet count 210 × 10^3^/mm^3^. The erythrocyte sedimentation rate was 57 mm/h and C-reactive protein was 24 mg/dl. Uric acid, C3, C4 components of complements, alanine aminotransferase, aspartate aminotransferase, sodium, potassium, urea, serum creatinine, and blood glucose were all within normal ranges. The urine examinations were positive for bacterial infection. Results revealed negative for autoantibodies, such as rheumatoid factor, antinuclear antibody, antineutrophil cytoplasmic antibody. Albumin was 20.6 g/L, serum IgG was 7.41 g/L and IgG index was 0.55 (Pasteur Cerba Laboratoire, France). Blood culture was positive twice for staphylococcus coagulase negative, and after antibiotic treatment resulted negative. Because of bacteremia and stiffness of the neck, the biochemical parameters of cerebrospinal fluid were performed: total proteins 0.51 g/l, albumin 236 mg/l, IgG 47 mg/l and isoelectric focusing (cerebrospinal fluid and serum); absence of anomaly of the IgG pattern in the cerebrospinal fluid and in the serum (Pasteur Cerba Laboratoire, France). Radiography of the chest showed pleuropneumonia, whereas those of the palms, talocrural and sacroiliac joints had no changes. We followed up on the patient for one year managing the constitutional symptoms, laboratory parameters and radiographic findings. During the follow-up period, the patient experienced many episodes of reactive arthritis approaching the disease chronic onset. After one year, pelvic radiography revealed asymmetric erosive sacroileitis on the right side (Figure 1).

Based on these findings, we assumed the diagnosis of poststaphylococcal reactive arthritis. Treatment was initiated with Vancomycin based on the antibiogram to cover bacteremia and pleuropneumonia, while NSAIDs and corticosteroids were used for poststaphylococcal reactive arthritis. Clinical, laboratory findings, and lung radiographic changes improved, and the blood culture became negative.

## Discussion

Several studies have reported the reactive arthritis after urogenital and enteric infections such as Salmonella, Shigella, Yersinia and Chlamidia, also after poststreptococcal infections, but staphylococcal infection very rarely triggers reactive arthritis [7]. Staphylococcus coagulase negative are present on the skin and mucosa as normal human flora but they can cause bacteremia, diverse prosthetic device-related infections, and urinary infection, whereas Staphylococcus epidermidis is a common cause of catheter-associated urinary tract infections [8]. Most, if not all, of triggering organisms produce lipopolysaccharide (LPS) and share a capacity to attack mucosal surfaces, to invade host cells, and to survive intracellulary [9,10]. We believe that this is a rare case of reactive arthritis developing after staphylococcal coagulase negative infection. While the detection of coagulase negative staphylococcus at the site of infection or in the bloodstream is not difficult by standard microbiological culture methods, interpretations of these results are frequently problematic. The minimum of two positive blood cultures are required to conclude bacteremia with coagulase negative staphylococcus, which was also our case. After treatment as described above, among other parameters, lung radiographic changes improved.

## Conclusion

We report a patient who developed reactive arthritis as a sequel to a staphylococcal coagulase negative infection, and physicians should be aware of possible reactive arthritis after staphylococcal coagulase negative bateremia.

## Abbreviations

HLA: human leukocyte antigen; IgG: immunoglobulin G; LPS: lypopolysaccharide; NSAID: non-steroidal anti-inflammatory drug; PIP: proximal interphalangeal.

## Consent

Written informed consent was obtained from the patient for publication of this case report and the accompanying images. A copy of the written consent is available for review by the Editor-in-Chief of this journal.

## Competing interests

The authors declare that they have no competing interests.

## Authors' contributions

XK analyzed and interpreted patient data and was a major contributor in writing the manuscript. SR analyzed and interpreted patient data. MG analyzed the data and contributed in writing the manuscript. BK analyzed and interpreted patient data and contributed in writing the manuscript. FA analyzed and interpreted patient data. DK contributed in writing the manuscript. All authors read and approved the final manuscript.

## References

[B1] Hannu T Inman R Granfors K Leirisalo-Repo M Reactive arthritis or post-infectious arthritis? Best Pract Res Clin 2006 20 419 433 10.1016/j.berh.2006.02.003 16777574

[B2] Pollanen R Sillat T Pajarinen J Levon J Kaivosoja E Konttinen YT Microbial antigens mediate HLA-B27 diseases via TRLs J Autoimmun 2009 32 172 177 10.1016/j.jaut.2009.02.010 19299108

[B3] Silva-Ramirez B Vargas-Alarcon G Granados J Burgos-Vargas R HLA antigens and juvenile onset spondyloarthritides: negative association with non-B27 alleles Clin Exp Rheumatolog 2005 23 721 723 16173256

[B4] Orden-Martinez B Martinez-Ruiz R Millan-Perez R What are we learning about Staphylococcus saprophyticus? Enferm Infecc Microbiol Clin 2008 26 6 495 499 10.1016/S0213-005X(08)72777-0 19094862

[B5] Piette A Verschraegen G Role of coagulase negative staphylococci in human desease Vet Microbiol 2009 134 1-2 45 54 10.1016/j.vetmic.2008.09.009 18986783

[B6] Bollow M Hermann KG Biedermann T Sieper J Schontube M Braun J Very early spondyloarthritis: where the inflammation in the sacroiliac joints starts Ann Rheum Dis 2005 64 1644 1646 10.1136/ard.2004.034967 16227415 PMC1755289

[B7] Siam AR Hammoudeh M Staphylococcus triggered reactive arthritis Ann Rheum Dis 1995 54 131 133 10.1136/ard.54.2.131 7702401 PMC1005536

[B8] Cho SH Naber K Hacker J Ziebuhr W Detection of the icaADBC gene cluster and biofilm formation in Staphylococcus epidermidis isolates from catheter-related urinary tract infections Int J Antimicrom Agents 2002 19 6 570 575 10.1016/S0924-8579(02)00101-2 12135850

[B9] Rupp ME Archer GL Coagulase negative staphylococci: pathogens associated with medical progress Clin Infect Dis 1994 19 2 231 243 7986894 10.1093/clinids/19.2.231

[B10] Pintens V Massonet C Merckx R Vandercastele S Peetermans WE Knobloch JK Van Eldere J The role of sigmaB, in persistence of Staphylococcus epidermidis foreign body infection Microbiology 2008 154 9 2827 2836 10.1099/mic.0.2007/015768-0 18757816

